# Indole 3-acetic acid, indoxyl sulfate and paracresyl-sulfate do not influence anemia parameters in hemodialysis patients

**DOI:** 10.1186/s12882-017-0668-5

**Published:** 2017-07-26

**Authors:** Stanislas Bataille, Marion Pelletier, Marion Sallée, Yvon Berland, Nathalie McKay, Ariane Duval, Stéphanie Gentile, Yosra Mouelhi, Philippe Brunet, Stéphane Burtey

**Affiliations:** 1 Centre de néphrologie et transplantation rénale, Assistance Publique des Hôpitaux de Marseille, Aix-Marseille University, Marseille, France; 2 Phocean Nephrology Institute, Clinique Bouchard, 77 rue du Docteur Escat, 13006 Marseille, France; 3 ELSAN, Clinique Bouchard, Marseille, France; 4 UMR_S 1076, Vascular Research Center of Marseille, INSERM, Aix Marseille University, Marseille, France; 5 Association des Dialysés Provence et Corse, Marseille, France; 6 EA3279, unité de recherche santé publique et maladies chroniques, Aix-Marseille University, Marseille, France

**Keywords:** Anemia, Chronic hemodialysis, Uremic toxins, Erythropoietin, Indoxyl sulfate, Indolic solutes

## Abstract

**Background:**

The main reason for anemia in renal failure patients is the insufficient erythropoietin production by the kidneys. Beside erythropoietin deficiency, in vitro studies have incriminated uremic toxins in the pathophysiology of anemia but clinical data are sparse. In order to assess if indole 3-acetic acid (IAA), indoxyl sulfate (IS), and paracresyl sulfate (PCS) -three protein bound uremic toxins- are clinically implicated in end-stage renal disease anemia we studied the correlation between IAA, IS and PCS plasmatic concentrations with hemoglobin and Erythropoietin Stimulating Agents (ESA) use in hemodialysis patients.

**Methods:**

Between June and July 2014, we conducted an observational cross sectional study in two hemodialysis center. Three statistical approaches were conducted. First, we compared patients treated with ESA and those not treated. Second, we performed linear regression models between IAA, IS, and PCS plasma concentrations and hemoglobin, the ESA dose over hemoglobin ratio (ESA/Hemoglobin) or the ESA resistance index (ERI). Third, we used a polytomous logistic regression model to compare groups of patients with no/low/high ESA dose and low/high hemoglobin statuses.

**Results:**

Overall, 240 patients were included in the study. Mean age ± SD was 67.6 ± 16.0 years, 55.4% were men and 42.5% had diabetes mellitus.

When compared with ESA treated patients, patients with no ESA had higher hemoglobin (mean 11.4 ± 1.1 versus 10.6 ± 1.2 g/dL; *p* <0.001), higher transferrin saturation (TSAT, 31.1 ± 16.3% versus 23.1 ± 11.5%; *p* < 0.001), less frequently an IV iron prescription (52.1 versus 65.7%, *p* = 0.04) and were more frequently treated with hemodiafiltration (53.5 versus 36.7%). In univariate analysis, IAA, IS or PCS plasma concentrations did not differ between the two groups.

In the linear model, IAA plasma concentration was not associated with hemoglobin, but was negatively associated with ESA/Hb (*p* = 0.02; *R* = 0.18) and with the ERI (*p* = 0.03; *R* = 0.17). IS was associated with none of the three anemia parameters. PCS was positively associated with hemoglobin (*p* = 0.03; *R* = 0.14), but negatively with ESA/Hb (*p* = 0.03; *R* = 0.17) and the ERI (*p* = 0.02; *R* = 0.19). In multivariate analysis, the association of IAA concentration with ESA/Hb or ERI was not statistically significant, neither was the association of PCS with ESA/Hb or ERI. Identically, in the subgroup of 76 patients with no inflammation (CRP <5 mg/L) and no iron deficiency (TSAT >20%) linear regression between IAA, IS or PCS and any anemia parameter did not reach significance.

In the third model, univariate analysis showed no intergroup significant differences for IAA and IS. Regarding PCS, the *Low Hb/High ESA* group had lower concentrations. However, when we compared PCS with the other significant characteristics of the five groups to the *Low Hb/high ESA* (our reference group), the polytomous logistic regression model didn’t show any significant difference for PCS.

**Conclusions:**

In our study, using three different statistical models, we were unable to show any correlation between IAA, IS and PCS plasmatic concentrations and any anemia parameter in hemodialysis patients. Indolic uremic toxins and PCS have no or a very low effect on anemia parameters.

## Background

Anemia is a major burden in renal failure. Within patients with chronic kidney disease (CKD) stage 2 to 5, anemia occurs in approximately 20% of patients but as the kidney function decreases its prevalence rises to more than 40% in patients with CKD stage 5 [1]. In the Dialysis Outcomes and Practices Patterns Study (DOPPS), around 90% of hemodialysis patients were treated with erythropoiesis stimulating agents (ESA) in 2011 [https://www.dopps.org]. Clinical practice guidelines for anemia in CKD have recently been published by the KDIGO [2].

The main reason for the defective erythropoiesis in renal failure patients is the relative deficiency in erythropoietin (EPO) production by the peritubular cells of kidneys [3]. Nevertheless, beside EPO deficiency, other factors have been incriminated in anemia: deficiencies in iron, folates and cobalamin, chronic inflammation, uncontrolled hyperparathyroidism, drugs including ACE inhibitors, or uremic toxins [2–4]. It is important to understand underlying mechanisms of anemia in CKD because the use of ESA might be at least ineffective in some patients or even deleterious in patients with high dosages [5].

The uremic toxins, *i.e.* harmful compounds accumulating during renal failure, have been incriminated in the pathophysiology of anemia in CKD: sera from uremic patients inhibit hematopoietic progenitor’s growth. However, clinical data in the field are sparse [6].

The role of indolic toxins or paracresyl-sulfate (PCS) in anemia has recently been proposed. Indoxyl sulfate (IS) and indole-3-acetic acid (IAA) are protein bound indolic uremic toxins derived from tryptophan. IS impairs erythropoiesis in an HIF dependent manner and suppresses the *EPO* gene transcription during hypoxia [7]. This indolic toxin as well as IAA, activates the transcription factor aryl hydrocarbon receptor (AhR) [8]. During hypoxia, HIF2α forms a heterodimer with ARNT (AhR nuclear transcriptor) and translocates in the nucleus leading to *EPO* gene expression. IS increases the AhR/ARNT complex in the nucleus and decreases the HIF2α/ARNT complex [9]. Another mechanism leading to anemia is induction of eryptosis by IS [10]. This mechanism has been proposed to favor anemia during renal failure.

Beside these in vitro results, the role of indolic toxins in anemia was also suggested by the use of AST120. AST120 is an oral sorbent of several compounds including indols. It decreases IS and PCS blood concentrations. Wu et al. showed in a recent clinical trial that AST120 decreased IS and PCS levels and improved hemoglobin level [11]. Nevertheless, to the best of our knowledge, no clinical study has linked anemia or ESA resistance to plasma concentrations of these specific uremic toxins.

In order to assess if IS, IAA and PCS are clinically implicated in end-stage renal disease anemia we studied the correlation between their plasmatic concentrations with hemoglobin and ESA use in hemodialysis patients.

## Methods

### Subjects and study design

Between June and July 2014, we conducted an observational cross sectional study in two hemodialysis centers in Marseille, France to analyze the correlation between IS, IAA and PCS plasmatic concentrations and the anemia parameters. The two centers were the Nephrology Dialysis and Renal Transplant Center, Assistance Publique des Hôpitaux de Marseille, and the Phocean Institute of Nephrology.

All patients on hemodialysis for more than 3 months and on either no ESA, or darbepoietin treatment were included in the study. In order to compare ESA dose, patient treated with other ESA’s than darbepoietin were excluded from the study. Pregnant women and patients aged <18 years were not included. According to French law, it is not necessary or possible to obtain approval from an ethics committee (Comité de Protection des Personnes, CPP) for this type of non-interventional study. Moreover, CPP ethics committees are not entitled to issue waivers of approval for this type of study.

### Clinical, biological, and hemodialysis parameters

Clinical and biological following data were recorded from the patient’s medical files: age, gender, nephropathy, time on dialysis, history of diabetes mellitus, body weight, biological parameters including hemoglobin, transferrin saturation (TSAT), ferritin, as well as predialysis solute concentrations as urea, creatinine, and β2-microglobulin, dialysis parameters and vascular access type, treatments including weekly ESA and IV iron doses. Most classical factors associated with ESA resistance as iron status, inflammation (c-reactive protein, CRP), or parathormone level (PTH) were included in the study [12].

Dialysis parameters (Kt/V and type of dialysis, *i.e.* hemodialysis or hemodiafiltration) were recorded at the mid-week session, and biological analyses were all performed at the beginning of hemodialysis session. Dialysis dose was estimated by a single-pool Kt/V (spKt/V), as recommended by Daugirdas et al. [13].

Because anemia in hemodialysis patients is influenced by ESA treatment and dose, two indexed dose-response markers were calculated. First, the ESA dose over hemoglobin ratio (ESA/Hb) was calculated as the weekly dose of darbepoietin (μg) divided by the hemoglobin plasma concentration (g/dL). Second, the ESA resistance index (ERI), a well-known ESA quantification marker associated with low survival in hemodialysis patients was defined as ESA/Hb divided by post-dialysis weight (kg) [12, 14–16]. In the two centers, IV iron (Ferric Hydroxide Saccharose 100 mg/5 mL, Mylan®, USA) prescription was based on TSAT and ferritin according to the physician’s decision.

### Uremic toxins dosage

Serum and plasma samples from patients were stored at −80 °C until use and determination of uremic solute. IS, IAA, and paracresyl sulfate were measured in serum by high performance liquid chromatography as previously described [17]. β2-microglobulin was measured by immuno-enzymology.

### Statistical analysis

Quantitative data were expressed as mean ± standard deviation (SD), whereas categorical data were expressed as frequency and percentage.

In order to compare characteristics in the two groups of patients: *With ESA* and *Without ESA*, univariate group comparisons were performed using Student T-test and chi-square tests.

To assess the correlation of toxins IS, IAA and PCS plasmatic concentrations with hemoglobin and ESA use in hemodialysis patients, two methods were used.

First, a multivariate Linear Regression model was used to estimate the relationship between Hb, ESA/Hb or ERI levels and the other characteristics. The β coefficients and two tailed *p*-values were performed. The level of significance was set at a *p*-value <0.05. This model was also performed in the subgroup of patients with no inflammation (CRP <5 mg/L) and no iron deficiency (TSAT >20%).

Then, to better analyze this correlation, we performed a Polytomous Logistic Regression model. We separated patients in 6 subgroups according to the hemoglobin level and the ESA treatment. For each hemoglobin level (high hemoglobin >10 g/dL and low hemoglobin ≤10 g/dL), we separated the ESA treatment into 3 categories: no treatment, low ESA dose <40 μg/week and high dose ≥40 μg/week. The 40 μg/week cutoff was chosen because it was the median dose within patients receiving ESA. In literature, hypo responsiveness to ESA is defined as a low Hb despite high ESA dose, thus, the *Low Hb/High ESA* group was taken as the reference group for the polytomous logistic regression model. We introduced into the multivariate model all the variables with a *p*-value <0.20 in the univariate model, then we performed a backward elimination procedure so as to conserve into the final model only the variables with a *p*-value < 0.05. The adjusted odd ratios (OR) values with their 95% confidence interval were performed, and the level of significance was set at a *p*-value <0.05. Statistical analysis was performed using Statistical Package for Social Sciences (SPSS) software (version 20, SPSS, Inc., Chicago, IL, USA).

## Results

Within the two hemodialysis centers, 240 patients were included in the study. Mean age ± SD was 67.6 ± 16.0 years, 55.4% were men and 42.5% had diabetes mellitus (Table 1). Mean time on dialysis was 82 ± 121 months.

**Table 1:** General characteristics and factors associated with ESA prescription: univariate analysis (Student T-test test or chi square test as appropriate, *n* = 240 patients)

	Total population (*n* = 240)	Patients without ESA (*n* = 71)	Patients with ESA (*n* = 169)	*p*-value
Age (years)	67.6 ± 16.0	68.6 ± 14.8	67.3 ± 16.6	0.56
Male gender	55.4%	62.0%	52.7%	0.19
Time on dialysis (months)	82 ± 121	80 ± 89	83 ± 133	0.87
Diabetes mellitus	42.5%	43.7%	42.0%	0.81
Dry weight (kg)	69.6 ± 14.9	71.4 ± 15.6	68.9 ± 14.6	0.23
ADPKD	4.6%	4.2%	4.7%	1.00
White cell count (G/L)	7.01 ± 4.62	6.55 ± 2.05	7.21 ± 5.34	0.31
Lymphocytes (G/L)	1.28 ± 0.58	1.34 ± 0.62	1.25 ± 0.57	0.27
Hemoglobin (g/dL)	10.8 ± 1.2	11.4 ± 1.1	10.6 ± 1.2	<0.001
MCV (fl)	94 ± 8	93 ± 6	95 ± 8	0.33
Platelet count (G/L)	203 ± 79	187 ± 70	209 ± 82	0.06
TSAT (%)	25.5 ± 13.5	31.1 ± 16.3	23.1 ± 11.5	<0.001
Ferritin (ng/mL)	530 ± 520	577 ± 640	510 ± 461	0.36
Albumin (g/L)	37.7 ± 5.3	37.9 ± 4.8	37.6 ± 5.5	0.65
β2 microglobulin (mg/L)	26.6 ± 7.4	27.4 ± 7.9	26.4 ± 7.1	0.40
Parathormone (pg/mL)	256 ± 318	263 ± 313	252 ± 119	0.82
Predialysis creatinine (μmol/L)	732 ± 253	748 ± 273	725 ± 244	0.52
Predialysis urea (mmol/L)	20.7 ± 6.6	20.4 ± 5.9	20.8 ± 6.8	0.64
C-reactive protein (mg/L)	20.0 ± 43.5	16.6 ± 37.8	21.4 ± 45.7	0.44
IAA (μmol/L)	4.73 ± 4.79	4.79 ± 5.06	4.71 ± 4.69	0.91
IS (μmol/L)	93.3 ± 51.5	91.1 ± 53.0	94.2 ± 51.0	0.67
PCS(μmol/L)	158 ± 99	165 ± 99	154 ± 99	0.44
ESA dose (μg/w)	-	-	53.9 ± 50.0	-
IV iron medication (%)	61.7%	52.1%	65.7%	0.04
Iron dose (mg/mo)	232 ± 267	173 ± 200	257 ± 288	0.03
Vascular access				
Native AV fistula	63.3%	54.9%	66.9%	0.17
AV graft	16.7%	18.3%	16.0%	
Catheter	20.0%	26.8%	17.2%	
HDF (%)	41.7%	53.5%	36.7%	0.02
Kt/V	1.51 ± 0.31	1.51 ± 0.26	1.51 ± 0.32	0.98

Results are provided as mean ± SD or percentages. *ESA* erythropoiesis stimulating agent, *ADPKD* autosomal dominant polycystic disease, *MCV* mean corpuscular volume, *IS* indoxylsulfate, *IAA* indole-3-acetic acid, *PCS* paracresyl-sulfate, *AV* arteriovenous, *HDF* hemodiafiltration

The etiology of the primary cause of renal failure was diabetic nephropathy in 23.8% of patients, vascular nephropathy in 20.4%, chronic interstitial nephritis in 15.4%, non-diabetic glomerular disease in 12.9%, autosomal dominant polycystic kidney disease in 4.6%, other in 6.7%, and unknown in 16.3%.

Clinical, dialysis and biological parameters are reported in Table 1. Most of patients (63.3%) had a native arteriovenous fistula, 20.0% a catheter and 16.7% an arteriovenous graft. Mean Kt/V was of 1.51 ± 0.31, which was in the recommended range of >1.2 [18]. Hemodiafiltration was the dialysis technique in 41.7% of patients, and conventional hemodialysis for the remaining 58.3%. Mean hemoglobin was 10.8 ± 1.2 g/dL, mean TSAT 25.5 ± 13.5% and mean ferritin 530 ± 520 ng/mL. Mean albumin was 37.7 ± 5.3 g/L. Mean IAA concentration was 4.73 ± 4.79 μmol/L; mean IS concentration 93.3 ± 51.5 μmol/L and mean PCS concentration 158 ± 99 μmol/L.

**Fig. 1:**
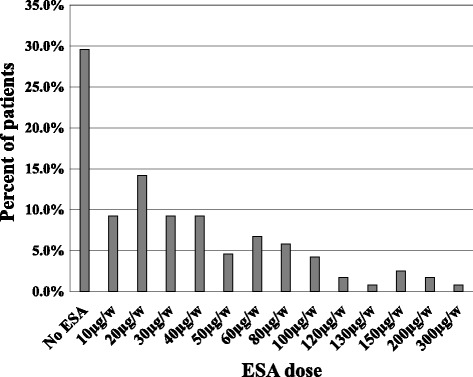
ESA weekly dose in hemodialysis patients (*n* = 240 patients)

**Fig. 2:**
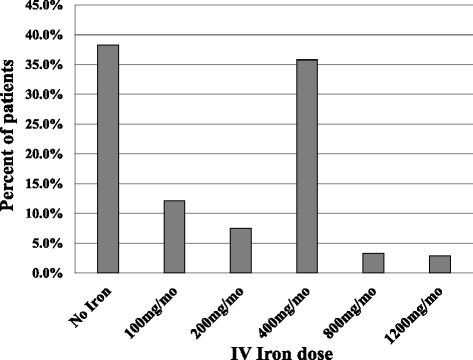
IV iron monthly dose in hemodialysis patients (*n* = 240 patients)

Regarding treatments, 29.5% (*n* = 71 patients) had no ESA and mean ESA dose in the treated group was 53.9 ± 50.0 μg per week (Table 1, Fig. 1); 61.7% of patients had IV iron supplementation and mean iron dose was of 232 ± 267 mg/month but most patients had either no iron, or 400 mg/month (i.e. 100 mg/week) (Table 1, Fig. 2).

When compared with ESA treated patients, patients with no ESA had higher hemoglobin (mean 11.4 ± 1.1 versus 10.6 ± 1.2 g/dL; *p*-value <0.001), higher TSAT (31.1 ± 16.3% versus 23.1 ± 11.5%; *p* < 0.001), less iron prescription (52.1% versus 65.7%, *p* = 0.04) and they were more frequently treated with hemodiafiltration (53.5% versus 36.7%) (Table 1). In univariate analysis, IAA, IS or PCS plasma concentrations did not differ between the two groups, thus, no multivariate model is provided.

**Fig. 3:**
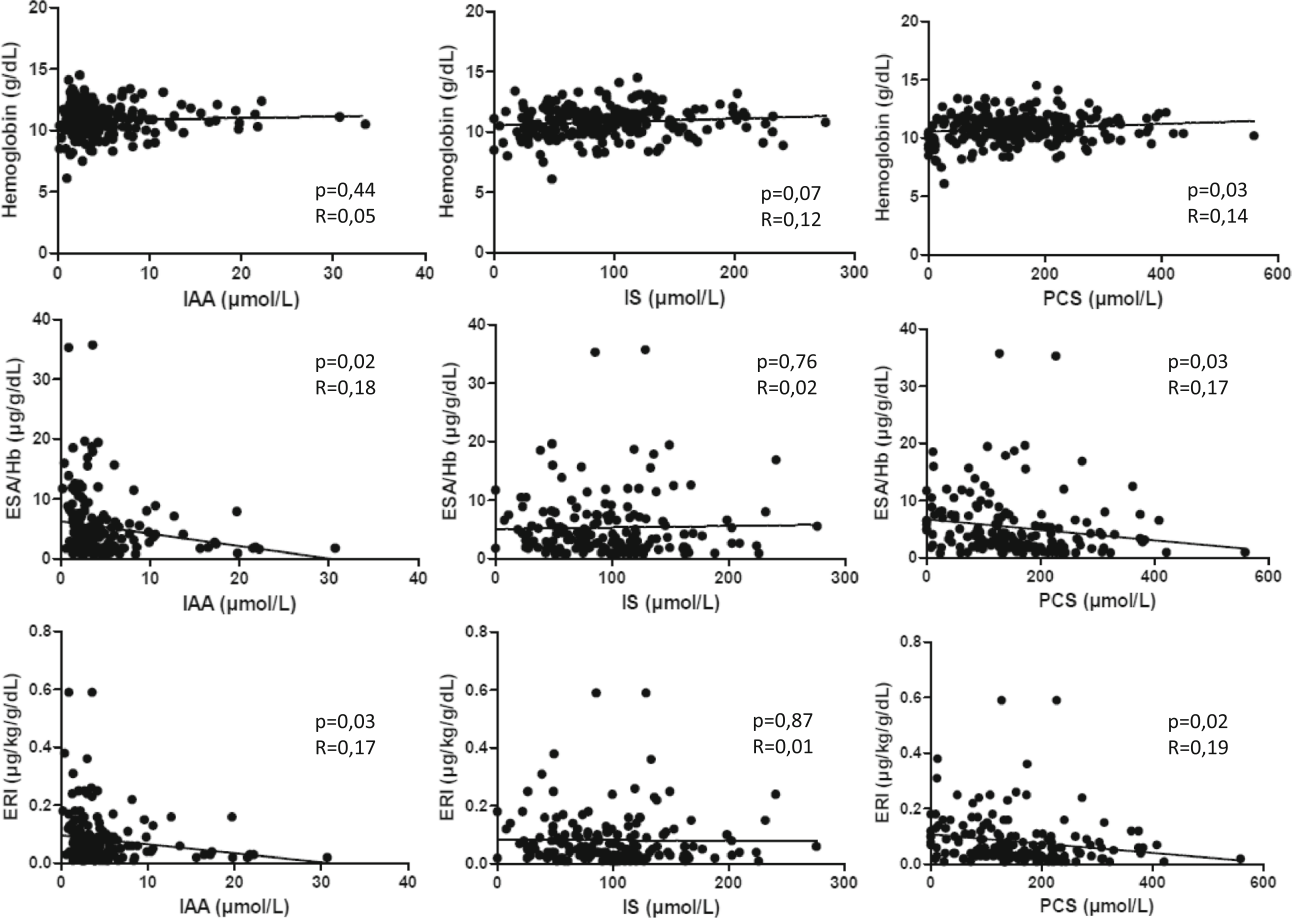
Association between Indole-3-acetic acid, Indoxyl Sulfate, and Paracresyl sulfate plasmatic concentrations and anemia parameters in hemodialysis patients (Linear regression, *n* = 240 patients). IAA: Indole-3-acetic acid; IS: Indoxyl Sulfate; PCS: Paracresyl sulfate; ESA/Hb: Erythropoietin Stimulating Agent weekly dose over hemoglobin ratio; ERI: Erythropoietin Stimulating Agent resistance index

Linear regression of IS, IAA and PCS with hemoglobin, ESA over hemoglobin ratio and ERI are reported in Fig. 3. IAA plasma concentration was not associated with crude hemoglobin concentration, but was negatively associated with ESA/Hb (*p* = 0.02; *R* = 0.18) and with the ERI (*p* = 0.03; *R* = 0.17). IS was not associated with any of the three anemia parameters. PCS was positively associated with hemoglobin (*p* = 0.03; *R* = 0.14), and negatively with ESA/Hb (*p* = 0.03; *R* = 0.17) and the ERI (*p* = 0.02; *R* = 0.19). In multivariate analysis, the association of IAA concentration with ESA/Hb and ERI was not statistically significant, neither was the association of PCS with ESA/Hb and ERI. The only factors which were associated with ESA/Hb and ERI in the multivariate analyses were age, TSAT, ferritin, albumin and the iron dose (Table 2). Only 76 patients had no inflammation (CRP <5 mg/L) and no iron deficiency (TSAT >20%). In this subgroup of patients, univariate linear regression of IS, IAA and PCS with Hb, ESA/Hb and ERI did not reach clinical significance (data not shown).

**Table 2:** Factors associated with ESA/Hb and ERI: multivariate analyses (Linear Regression Model, *n* = 240 patients)

	ESA/Hb (μg/g/dL)		ERI (μg/kg/g/dL ×10)	
	Coefficient (95% CI)	*p*-value	Coefficient (95% CI)	*p*-value
Age (per 10 years)	−0.47 [−0.66; −0.28]	0.02	−0.09 [−0.12; −0.06]	0.006
TSAT	−0.10 [−0.13; −0.07]	<0.001	−0.02 [−0.02; −0.01]	<0.001
Ferritin (per 10 ng/mL)	0.02 [0.03; 0.01]	0.003	0.004 [0.003; 0.005]	0.002
Albumin	−0.27 [−0.33; −0.21]	<0.001	−0.05 [−0.06; −0.04]	<0.001
Iron dose	0.003 [0.002; 0.004]	0.004	0.001 [0.001; 0.001]	0.007

*ESA/Hb* ESA dose over hemoglobin ratio, *ERI* ESA resistance index, *TSAT* transferrin saturation

To accurately analyze the link between anemia and uremic toxins, patients were separated in 6 subgroups according to the hemoglobin level and the ESA treatment as described in the methods section. Frequencies and main biological parameters are reported in Table 3. In the univariate analysis, no intergroup significant difference was found for IAA and IS. Regarding PCS, the *Low Hb*/*High ESA* group had lower concentrations. However, when we compared PCS with the other significant characteristics of the five groups to the low *Low Hb*/*High ESA* (our reference group), the polytomous logistic regression model didn’t show any significant difference for PCS. In this latter, only ferritin levels, TSAT and albumin differed between groups as reported in Table 4.

**Table 3:** Factors associated with high or low hemoglobin level and ESA dose (ANOVA or Chi-square test, *n* = 240 patients)

	Hb >10 g/dL			Hb ≤10 g/dL			*p*-value
	no ESA (*n* = 63)	ESA ≤40 μg/w (*n* = 75)	ESA >40 μg/w (*n* = 41)	no ESA (*n* = 8)	ESA ≤40 μg/w (*n* = 25)	ESA >40 μg/w (*n* = 28)	
Age (years)	67.8 ± 15.3	67.1 ± 16.2	69.9 ± 15.4	74.5 ± 8.1	64.8 ± 17.6	65.9 ± 18.3	0.63
Gender (male)	61.9%	58.7%	41.5%	62.5%	64.0%	42.9%	0.19
Diabetes	44.4%	41.3%	43.9%	37.5%	48.0%	35.7%	0.96
Hemoglobin (g/dL)	11.6 ± 0.9	11.2 ± 0.8	11.1 ± 0.8	9.6 ± 0.6	9.5 ± 0.4	9.0 ± 0.9	<0.001
TSAT (%)	29.2 ± 13.5	23.5 ± 9.2	22.7 ± 11.1	45.8 ± 27.4	25.6 ± 9.8	20.1 ± 17.4	<0.001
Ferritin (ng/mL)	480 ± 330	426 ± 285	473 ± 304	1341 ± 1541	636 ± 314	677 ± 901	<0.001
Albumin (g/L)	38.4 ± 4.4	39.2 ± 4.1	37.5 ± 4.8		37.8 ± 4.6	33.0 ± 7.8	<0.001
β2 microglobulin (mg/L)	26.2 ± 7.0	25.7 ± 6.9	27.4 ± 8.2	34.1 ± 6.4	25.6 ± 6.2	27.4 ± 7.0	0.008
Parathormone (pg/mL)	268 ± 318	284 ± 315	195 ± 207	35.8 ± 9.8	264 ± 354	237 ± 438	0.83
Predialysis creatinine (μmol/L)	764 ± 282	769 ± 242	684 ± 250	224 ± 299	757 ± 220	638 ± 239	0.08
Predialysis urea (mmol/L)	20.8 ± 5.9	21.7 ± 6.7	20.0 ± 6.5	623 ± 153	21.0 ± 6.1	19.6 ± 8.3	0.39
C-reactive protein (mg/L)	12.2 ± 18.9	12.7 ± 28.4	26.0 ± 67.4	17.2 ± 5.5	23.1 ± 49.9	36.5 ± 37.1	0.02
IAA (μmol/L)	5.10 ± 5.28	5.73 ± 5.97	3.51 ± 3.50	51.9 ± 97.3	4.67 ± 3.05	3.78 ± 2.60	0.09
IS (μmol/L)	92.8 ± 54.7	97.9 ± 45.2	100.9 ± 49.9	2.29 ± 1.30	85.2 ± 55.5	82.3 ± 61.9	0.54
PCS(μmol/L)	169 ± 101	172 ± 94	150 ± 97	78.1 ± 37.6	175 ± 98	96 ± 99	0.01
Iron medication							
IV iron medication	54.0%	68.0%	73.2%	135 ± 75	48.0%	64.3%	0.10
Iron dose (mg/mo)	175 ± 188	228 ± 245	351 ± 318	163 ± 292	124 ± 167	314 ± 374	0.002
HDF (%)	54.0%	30.7%	31.7%	50.0%	52.0%	46.4%	0.05
Kt/V	1.51 ± 0.26	1.50 ± 0.28	1.50 ± 0.42	1.46 ± 0.30	1.57 ± 0.26	1.51 ± 0.34	0.95

Results are provided as mean ± SD or percentages. *Hb* Hemoglobin, *ESA* Erythropoietin stimulating agent, *TSAT* transferrin saturation, *IAA* indole-3-acetic acid, *IS* indoxylsulfate, *PCS* paracresyl-sulfate, *HDF* hemodiafiltration

**Table 4:** Polytomous logistic regression: factors associated with hemoglobin level or ESA requirement (*n* = 240 patients)

Predictor variables		*OR (95% CI)*	*p-value*
Hb > 10 g/dL no ESA	TSAT (%)	1.1 [1.06–1.2]	<0.001
	Ferritin (ng/mL)	0.9 [0.8–0.9]	<0.001
	Albumin (g/L)	1.1 [1.04–1.2]	0.01
Hb ≤ 10 g/dL no ESA	TSAT (%)	1.1 [1.05–1.2]	<0.001
	Ferritin (ng/mL)	0.9 [0.9–1.0]	0.06
	Albumin (g/L)	1.0 [0.9–1.2]	0.5
Hb > 10 g/dL ESA low dose	TSAT (%)	1.0 [1.01–1.1]	0.01
	Ferritin (ng/mL)	0.9 [0.8–0.9]	0.006
	Albumin (g/L)	1.2 [1.1–1.3]	<0.001
Hb ≤ 10 g/dL ESA low dose	TSAT (%)	1.0 [1.01–1.1]	0.05
	Ferritin (ng/mL)	0.9 [0.9–1.0]	0.2
	Albumin (g/L)	1.1 [1.02–1.2]	0.01
Hb ≤ 10 g/dL ESA high dose	TSAT (%)	1.06 [0.9–1.1]	0.07
	Ferritin (ng/mL)	0.9 [0.9–1.0]	0.05
	Albumin (g/L)	1.1 [1.01–1.2]	0.02

*The reference group is: Hb ≤ 10 g/dL ESA high dose*

## Discussion

In this observational multicentric study including 240 hemodialysis patients, we report no correlation between three protein bound uremic toxins -IAA, IS, and PCS- and anemia or ESA responsiveness. Three types of analyses were performed: first, we compared patients treated with ESA and those not treated. In this analysis, no correlation was found with the uremic toxins concentrations in the univariate model. Second, IAA, IS and PCS concentrations were analyzed as continuous variables. In the multivariate model, no link was found between these concentrations and Hb, ESA/Hb or even ERI. Finally, patients were divided in subgroups according to their anemia/ESA status and there again, no link was found between the studied uremic toxins and anemia in this population.

Many in vitro data plead for an effect of uremic toxins on erythropoiesis. Since years, it is well known that there is a dose dependent inhibition cultured primary bone marrow cell growth when serum extracted from renal failure patients is added to the milieu [19, 20]. Neither urea, nor creatinine were incriminated because they do not show this effect [3]. Indolic solutes, especially IS have been incriminated in anemia. Recently, Chiang et al. have reported that IS reduces EPO production in hypoxic condition by suppressing HIF activation and subsequent EPO transcription [7]. These results were confirmed in an independent study [21]. Recently the effect of IS on HIF in response to ischemia was demonstrated [22] as well as the key role of AhR in IS effect on EPO transcription under hypoxic condition [9]. These non-human experiments were important in vitro arguments on the role of indolic uremic toxins on anemia.

To the best of our knowledge, only one clinical study has focused on the correlation between IS level and anemia until now. This study failed to identify a relation between IS concentration and anemia in patients on peritoneal dialysis except in the non anuric patient’s subgroup in which IS concentration was correlated with Hb level [23].

IS and PCS derive from the metabolism of tryptophan and tyrosin respectively by bacteria within the gastrointestinal tract [24]. Hemodialysis patients without colon have very low IS and PCS plasma concentrations, demonstrating an important role of the gut in some uremic toxins metabolism [25]. Plasma concentrations of these toxins in renal failure patients are influenced by dietary intakes [26] or the use of sorbents as AST-120 [27]. In our study, IAA and PCS were associated with the ESA/Hb ratio or the ERI in univariate analysis, but these associations were no longer significant when adjusted on other parameters including albumin, an important nutritional maker. Parameters independently associated with anemia parameters in hemodialysis patients were TSAT, ferritin, and albumin. These parameters are the classical parameters influencing ESA sensibility [12]. Other parameters as CRP, β2-microglobulin or PTH did not reach significance in our study.

AST120 -an oral sorbent of uremic toxins- improved hemoglobin level in non-dialyzed CKD stage 5 patients when compared to placebo although ESA doses remained identical in the two groups [11]. This improvement was attributed to IS and PCS which plasmatic levels were lower in the AST-120 group. Nevertheless, AST120 is a non-specific adsorbent and its effect cannot definitely be attributed to these toxins removal because it could be due to other adsorbed compounds.

It is not clear why IS, IAA and PCS exert an important effect in vitro and no or a very low effect in vivo, but an important clue is that in vitro experiments are often performed with higher uremic toxin concentrations and there might be a dose effect relationship between toxin concentrations and anemia [7, 21].

Our study exerts some limits. First, we did not measure free concentration of IS and PCS; however, effects of IS in vitro is not influenced by albumin concentration in the media [8], and in clinical studies the observed concentrations of total and free IS are well correlated. Second, we studied prevalent hemodialysis patients in which Hb was performed every week and anemia very cautiously treated. Results in incident hemodialysis or in non-dialysis patients cannot be extrapolated from these results.

## Conclusion

In conclusion, we were unable to show any correlation between IAA, IS and PCS plasmatic concentrations and any anemia parameter in hemodialysis patients. It is likely that indolic uremic toxins and PCS have no or a very low effect on anemia parameters, i.e. Hb concentrations or ESA hypo responsiveness in this population.

## Abbreviations

AhR: Aryl hydrocarbon receptor
ARNT: AhR nuclear transcriptor
CKD: Chronic kidney disease
DOPPS: Dialysis outcomes and practices patterns study
EPO: Erythropoietin
ERI: ESA resistance index
ESA: Erythropoiesis stimulating agents
IAA: Indole-3-acetic acid
IS: Indoxyl sulfate
PCS: Paracresyl-sulfate
PTH: Parathormone
TSAT: Transferrin saturation
