# Publisher Correction: Indole 3-acetic acid, indoxyl sulfate and paracresyl-sulfate do not influence anemia parameters in hemodialysis patients

**DOI:** 10.1186/s12882-017-0716-1

**Published:** 2017-10-04

**Authors:** BMC Nephrology

**Affiliations:** London, UK

## Erratum

In the original publication of this article [1], Table 3 is incorrect, several cell values in column 4 (no ESA (*n* = 8)) have been incorrectly shifted during typesetting.

In this Erratum the correct and incorrect version of Table 3 are published.

The publisher apologizes for the inconvenience caused by this error to the authors and readers.

**Table 1:** Incorrect version of Table 3 as published on 26 July 2017. The incorrect cells are displayed in *italics*

	Hb >10 g/dL			Hb ≤10 g/dL			*p*-value
	no ESA (*n* = 63)	ESA ≤40 μg/w (*n* = 75)	ESA >40 μg/w (*n* = 41)	no ESA (*n* = 8)	ESA ≤40 μg/w (*n* = 25)	ESA >40 μg/w (*n* = 28)	
Age (years)	67.8 ± 15.3	67.1 ± 16.2	69.9 ± 15.4	74.5 ± 8.1	64.8 ± 17.6	65.9 ± 18.3	0.63
Gender (male)	61.9%	58.7%	41.5%	62.5%	64.0%	42.9%	0.19
Diabetes	44.4%	41.3%	43.9%	37.5%	48.0%	35.7%	0.96
Hemoglobin (g/dL)	11.6 ± 0.9	11.2 ± 0.8	11.1 ± 0.8	9.6 ± 0.6	9.5 ± 0.4	9.0 ± 0.9	<0.001
TSAT (%)	29.2 ± 13.5	23.5 ± 9.2	22.7 ± 11.1	45.8 ± 27.4	25.6 ± 9.8	20.1 ± 17.4	<0.001
Ferritin (ng/mL)	480 ± 330	426 ± 285	473 ± 304	*1341 ± 1541*	636 ± 314	677 ± 901	<0.001
Albumin (g/L)	38.4 ± 4.4	39.2 ± 4.1	37.5 ± 4.8		37.8 ± 4.6	33.0 ± 7.8	<0.001
β2 microglobulin (mg/L)	26.2 ± 7.0	25.7 ± 6.9	27.4 ± 8.2	*34.1 ± 6.4*	25.6 ± 6.2	27.4 ± 7.0	0.008
Parathormone (pg/mL)	268 ± 318	284 ± 315	195 ± 207	*35.8 ± 9.8*	264 ± 354	237 ± 438	0.83
Predialysis creatinine (μmol/L)	764 ± 282	769 ± 242	684 ± 250	*224 ± 299*	757 ± 220	638 ± 239	0.08
Predialysis urea (mmol/L)	20.8 ± 5.9	21.7 ± 6.7	20.0 ± 6.5	*623 ± 153*	21.0 ± 6.1	19.6 ± 8.3	0.39
C-reactive protein (mg/L)	12.2 ± 18.9	12.7 ± 28.4	26.0 ± 67.4	*17.2 ± 5.5*	23.1 ± 49.9	36.5 ± 37.1	0.02
IAA (μmol/L)	5.10 ± 5.28	5.73 ± 5.97	3.51 ± 3.50	*51.9 ± 97.3*	4.67 ± 3.05	3.78 ± 2.60	0.09
IS (μmol/L)	92.8 ± 54.7	97.9 ± 45.2	100.9 ± 49.9	*2.29 ± 1.30*	85.2 ± 55.5	82.3 ± 61.9	0.54
PCS(μmol/L)	169 ± 101	172 ± 94	150 ± 97	*78.1 ± 37.6*	175 ± 98	96 ± 99	0.01
Iron medication							
IV iron medication	54.0%	68.0%	73.2%	*135 ± 75*	48.0%	64.3%	0.10
Iron dose (mg/mo)	175 ± 188	228 ± 245	351 ± 318	163 ± 292	124 ± 167	314 ± 374	0.002
HDF (%)	54.0%	30.7%	31.7%	50.0%	52.0%	46.4%	0.05
Kt/V	1.51 ± 0.26	1.50 ± 0.28	1.50 ± 0.42	1.46 ± 0.30	1.57 ± 0.26	1.51 ± 0.34	0.95

**Table 2:** Correct version of Table 3, the corrected cells are displayed in **bold**

	Hb >10 g/dL			Hb ≤10 g/dL			*p*-value
	no ESA (*n* = 63)	ESA ≤40 μg/w (*n* = 75)	ESA >40 μg/w (*n* = 41)	no ESA (*n* = 8)	ESA ≤40 μg/w (*n* = 25)	ESA >40 μg/w (*n* = 28)	
Age (years)	67.8 ± 15.3	67.1 ± 16.2	69.9 ± 15.4	74.5 ± 8.1	64.8 ± 17.6	65.9 ± 18.3	0.63
Gender (male)	61.9%	58.7%	41.5%	62.5%	64.0%	42.9%	0.19
Diabetes	44.4%	41.3%	43.9%	37.5%	48.0%	35.7%	0.96
Hemoglobin (g/dL)	11.6 ± 0.9	11.2 ± 0.8	11.1 ± 0.8	9.6 ± 0.6	9.5 ± 0.4	9.0 ± 0.9	<0.001
TSAT (%)	29.2 ± 13.5	23.5 ± 9.2	22.7 ± 11.1	45.8 ± 27.4	25.6 ± 9.8	20.1 ± 17.4	<0.001
Ferritin (ng/mL)	480 ± 330	426 ± 285	473 ± 304	**1341 ± 1541**	636 ± 314	677 ± 901	<0.001
Albumin (g/L)	38.4 ± 4.4	39.2 ± 4.1	37.5 ± 4.8	**34.1 ± 6.4**	37.8 ± 4.6	33.0 ± 7.8	<0.001
β2 microglobulin (mg/L)	26.2 ± 7.0	25.7 ± 6.9	27.4 ± 8.2	**35.8 ± 9.8**	25.6 ± 6.2	27.4 ± 7.0	0.008
Parathormone (pg/mL)	268 ± 318	284 ± 315	195 ± 207	**224 ± 299**	264 ± 354	237 ± 438	0.83
Predialysis creatinine (μmol/L)	764 ± 282	769 ± 242	684 ± 250	**623 ± 153**	757 ± 220	638 ± 239	0.08
Predialysis urea (mmol/L)	20.8 ± 5.9	21.7 ± 6.7	20.0 ± 6.5	**17.2 ± 5.5**	21.0 ± 6.1	19.6 ± 8.3	0.39
C-reactive protein (mg/L)	12.2 ± 18.9	12.7 ± 28.4	26.0 ± 67.4	**51.9 ± 97.3**	23.1 ± 49.9	36.5 ± 37.1	0.02
IAA (μmol/L)	5.10 ± 5.28	5.73 ± 5.97	3.51 ± 3.50	**2.29 ± 1.30**	4.67 ± 3.05	3.78 ± 2.60	0.09
IS (μmol/L)	92.8 ± 54.7	97.9 ± 45.2	100.9 ± 49.9	**78.1 ± 37.6**	85.2 ± 55.5	82.3 ± 61.9	0.54
PCS(μmol/L)	169 ± 101	172 ± 94	150 ± 97	**135 ± 75**	175 ± 98	96 ± 99	0.01
Iron medication							
IV iron medication	54.0%	68.0%	73.2%	**37.5%**	48.0%	64.3%	0.10
Iron dose (mg/mo)	175 ± 188	228 ± 245	351 ± 318	163 ± 292	124 ± 167	314 ± 374	0.002
HDF (%)	54.0%	30.7%	31.7%	50.0%	52.0%	46.4%	0.05
Kt/V	1.51 ± 0.26	1.50 ± 0.28	1.50 ± 0.42	1.46 ± 0.30	1.57 ± 0.26	1.51 ± 0.34	0.95
